# Growth performance and carcass traits of indigenous Nigerian guinea fowl fed on different dietary protein levels

**DOI:** 10.5455/javar.2023.j693

**Published:** 2023-09-24

**Authors:** Olayinka Olubunmi Alabi, Cyril Abang, Olasunkanmi Peter Olajide, Rasaq Adekunle Animashahun, Stephen Otu Etta-Oyong

**Affiliations:** 1 Department of Animal Science, College of Agricultural Sciences, Landmark University Omu-Aran, Omu-Aran, Nigeria; 2 Department of Agricultural Extension, College of Agricultural Sciences, Landmark University Omu-Aran, Omu-Aran, Nigeria

**Keywords:** Carcass, growth, indigenous, keet, protein

## Abstract

**Objective:**

This work examined the results of different dietary protein levels on indigenous Nigerian guinea fowl growth performance and carcass yield.

**Materials and Methods:**

One hundred and eight unsexed indigenous keets were randomly assigned to three treatments with experimental diets containing 22%, 24%, and 26% crude protein (CP) in a completely randomized design, with 3 replicates with 12 keets in each replicate. The parameters were measured, including proximate composition, feed intake, growth rate, feed conversion ratio (FCR), and carcass yield.

**Results:**

The birds fed diets containing 24% CP gained the most body weight at 619.83 gm and had the highest FCR of 3.45. The presence of CP had an impact that was significant (*p *< 0.05) on most carcass traits and prime cuts but not on gastrointestinal tract weight, head weight, dress percentage, or organ yield.

**Conclusion:**

The study concluded that the dietary CP level of 24% was optimal for body weight gain and carcass yield.

## Introduction

Even though these birds evolved in Africa and have a ready market on the continent, guinea fowl (*Numida meleagris*) yield is still in its early stages [1]. In Nigeria, they are second only to domestic chickens in terms of number and protein supply [2]. The northern Guinea savanna zone contains a large population of guinea fowl, but the forest-dwelling crested guinea fowl can also be found in the southern region. The number of partially tended guinea fowl in Nigeria is estimated to be over 50 million and is widely scattered throughout the country’s savanna areas [3], making up about 25% of Nigeria’s total poultry population and a socially acceptable source of animal protein [4]. According to Oke et al. [1], these birds have high socioeconomic importance in remote areas, where they are primarily raised to provide meat and eggs and a means of income for local farmers. Guinea fowls are left to rummage for worms, insects, seeds, leaves, and fruits throughout farmsteads, and as a result, their productivity is low [5].

Guinea fowl possess a higher protein content, lower carcass fat, and more iron and vitamin E than domestic fowl [6]. There is a scarcity of data on indigenous guinea fowl production, which slows the industry’s growth despite increasing demand [5,7]. Understanding the degree of feed protein, these birds need is necessary to efficiently utilize local poultry resources to boost poultry output. Hence, the goal of this research was to investigate different protein levels in the guinea fowl diet to provide farmers and other stakeholders with a standardized protein requirement for guinea fowl farming.

## Materials and Methods

### Ethical approval

All birds used in this study and their management have been authorized by the Landmark University Animal Care and Use Committee (LUAC/2021/0018A).

### The experimental site

The Poultry Unit of Landmark University‘s Teaching and Research Farm in Omu-Aran was used for this study. One hundred and eight unsexed indigenous day old keets hatched in Landmark University‘s Animal Science laboratory were assigned randomly to 3 treatments after brooding, each of which was replicated 3 times in a completely randomized design with 12 keets in each replicate over 13 weeks. Diets were designed to provide the necessary protein for optimal performance, with treatments one (T1), two (T2), and three (T3) having a protein content of 22%, 24%, and 26%, indicating a lower, medium, and upper thresholds, respectively.

### Data collection

The Association of Official Analytical Chemists [8] methodologies were used to determine the proximate composition of feed and fecal samples collected for apparent digestibility studies. The guinea fowls‘ body weights were measured weekly on a digital scale (Ohaus, model PA512). The difference between the feed delivered and the feed left over after each session was used to calculate the birds‘ feed intake (FI). The feed conversion ratio (FCR) was calculated by dividing the weekly FI per replicate by the weight gain of the birds.

### The estimation of carcass traits

Two birds from each replicate of the experimental treatment were chosen at 13 weeks. These birds were slaughtered after being taken off feed for 12 h. The birds chosen had live weights closer to the group‘s average. The birds‘ jugular veins were cut and bled, and then their feathers were softened by soaking them in hot water for around 2 min before being hand-picked. After removing the shanks, head, neck, and viscera, the dressed weight was taken.

### Statistical analyses

A one-way analysis of variance was employed in analyzing all the data collected. Internal organs and carcasses were expressed as a percentage of live weight. Duncan’s new multiple range test of GENSTAT Release 10.3DE [9] software programs was used to distinguish means when they were statistically significant.

## Results

### Feed formulation

The feed formulated for the experiment is shown in Table 1. The metabolizable energy is isocaloric for all the treatments (2,650 kcal/kg), while the crude protein (CP) levels were 22%, 24%, and 26%, respectively. The CP levels were such that there was a low, middle, and upper thresholds that would be sufficient for the birds‘ optimum performance.

### Growth performance

Table 2 shows the growth metrics of indigenous guinea fowl (*N. meleagris*). The effects of varying CP levels were not significantly different (*p *> 0.05) on the parameters measured. T2 had a higher value (564.67 gm) for total weight gain than the values (530.08 and 516.91 gm) of T1 and T3 (26%) CP, respectively. T2 also had the highest daily weight gain (6.20 gm) value. Furthermore, T3 has the highest value (1,953.12 gm) for total FI, while T2 and T1 had a close range (1,933.13 and 1,929.52 gm, respectively). For daily FI, T3 had the highest value of 21.46 gm. The FCR in all three treatments was insignificant (*p *> 0.05), with T2 having the best FCR of 3.45.

### Carcass evaluation

The CP level in the treatments significantly (*p *< 0.05) influenced the carcass trait and prime cut of indigenous guinea fowls, as indicated in Table 3. The effect of protein inclusion in the diets was insignificant (*p *> 0.05) on gastrointestinal weight, head weight, or dress percentage, but T2 had greater values for carcass metrics obtained. The effect of the protein inclusion level in the diet was insignificant (*p *> 0.05) on the weight of the organs evaluated. T3 had the highest values for gizzard weight (22.46 gm), lung weight (7.51 gm), proventriculus (2.37 gm), and small intestine weight (17.60 gm), whereas T2 had the highest values for heart weight (3.24 gm) and crop weight (4.02 gm).

## Discussion

### Effect of the varying protein on growth performance

The effect of dietary treatments on weight gain, daily FI, and FCR was shown to be significant (*p *< 0.05) in this work [1]. The results revealed that birds fed a 24% CP diet gained the highest weight of 619.83 gm [1]. However, Singh et al.’s [10] finding of lower mean weight (429.0 gm) contradicts this finding. In addition, birds fed a 22% CP diet had a slightly lower final mean weight of 586.80 gm, while those fed a 26% CP diet had the lowest (566.43 gm) weight [1]. This finding agrees with Amoah et al. [11] and Rafiu et al. [12], who stated that an increment in dietary protein intake did not impact guinea fowl weight gain. The highest weight reported in this work, however, was not up to that obtained by Khairunnesa et al. [13] during week 13 of their study. This difference could be attributed to environmental factors [1]. A bird’s genetic makeup, nutrition, hormones, tissue-specific regulatory mechanisms, and environmental influences are only a few factors that might influence growth [1].

This study evaluated the FI of keets fed three diets—T1, T2, and T3—with CP levels of 26%, 24%, and 22%, respectively. Keets fed T3 ingested the most feed (1,953.12 gm), followed by those fed T2 (1,933.13 gm) and those fed T1 (1,929.52 gm) [1]. Amoah et al. [11] reported a 1,770 gm FI in a similar trial, which is lower than the present findings. Alabi et al. [14] pointed out that poultry birds consume more feed when their energy requirements are unmet, which explains the higher feed consumption in keets. This shows that the T3 diet was possibly energy-restrictive, resulting in the keets consuming more feed than those on T1. In this study, the FI of keets fed three different diets—T1, T2, and T3—with corresponding CP levels of 26%, 24%, and 22% was examined. Out of all of these diets, keets on T3 consumed the most feed (1,953.12 gm), followed by keets on T2 (1,933.13 gm) and keets on T1 (1,929.52 gm) [1]. These results are higher than those of Amoah et al. [11], who reported a 1,770 gm feed consumption in a related trial. Alabi et al.’s [14] assertion that birds tend to consume more feed when their energy requirements are not met explains the higher feed consumption observed in keets. This implies that the T3 diet may have been energy-restrictive, causing the keets to consume more feed than those on other diets [15].

**Table 1.** Feed composition for keet phase.

Ingredients	T1	T2	T3
Maize	41	41	38
SBM	25	30	37
GNC	13.5	14	14
Corn bran	7.5	2	1
Wheat offal	9	9	6
Bone meal	2	2	2
Limestone	2	2	2
Salt	0.25	0.25	0.25
Lysine	0.25	0.25	0.25
Methionine	0.25	0.25	0.25
Premix	0.25	0.25	0.25
Enzyme	0.1	0.1	0.1
Vitamin E	0.1	0.1	0,1
Total	100	100	100
Calculated			
Metabolizable energy (kcal/kg)	2,650	2,650	2,650
CP (%)	22	24	26

SBM = soya bean meal; GNC = groundnut cake.

The best performance in terms of feed conversion efficiency was in the 24% CP diet, with a ratio of 3.45. In contrast to the results of the present study, the study by Batkowska et al. [16] reported a less efficient FCR of 5.28. It is crucial to keep in mind that elements like nutrition and environmental factors may have an impact on these variations. Protein is an important nutrient. It affects the growth and health status of the guinea fowl significantly.

The higher body weight of guinea fowl fed the 24% CP diet may indicate that the birds’ nutritional requirements were met to a greater extent [12]. The improved FCR of this diet was responsible for the increase in overall weight gain. Hence, it is vital to take the CP level into account while formulating the diet to maximize guinea fowl growth and feed efficiency.

### The effect of CP on carcass traits

Table 3 shows the effect of CP on the carcass traits of the guinea fowl birds in T1 (22% CP), T2 (24% CP), and T3 (26% CP), respectively.

#### Live weight

When assessing the impact of incorporating CP in animal feed, live weight is an essential indicator. The impact of CP inclusion on live weight was examined in this study, and it was observed that the mean live weight value was significant (*p *> 0.05). T2 had the highest live weight value of 654.50 gm across the treatments. This figure is higher than the live weight of 572.54 gm recorded by Amoah et al. [11] at 11 weeks. However, studies have reported different live weight values at different time points. At week 14, for example, Ebegbulem and Asuquo [3] reported a live weight value of 975.0 gm, whereas Chiroque et al. [17] recorded a significantly higher value of 1,141.82 gm. The differences observed in these experiments could be attributable to various factors, including the age of the scavenging guinea fowls and the environmental conditions under which the experiments were carried out.

**Table 2.** Growth performance of guinea fowl (*N. meleagris*).

Parameters	Treatments			SEM
	T_1_ (22%)	T_2_ (24%)	T_3_ (26%)	
Initial weight	56.7	55.16	49.52	1.87
Final weight	586.80	619.83	566.43	16.49
TWG	530.08	564.67	516.91	16.02
ADG	5.82	6.20	5.68	.18
TFI	1,929.52	1,933.13	1,953.12	5.72
DFI	21.20	21.24	21.46	.06
FCR	3.67	3.45	3.79	.11
Mortality	.00	.00	0.31	.10

**Table 3.** Carcass traits of guinea fowl (*N. meleagris*).

Treatments	Parameters (gm)			
	T_1_ (22%)	T_2_ (24%)	T_3_ (26%)	SEM
Live weight	637.83^a^	654.50^a^	519.33^b^	16.82
Hot carcass weight	628.83^a^	647.83^a^	511.83^b^	16.79
Featherweight	55.83^a^	56.50^a^	32.33^b^	3.10
De-feather weight	573.00^a^	591.33^a^	479.50^b^	14.09
GIT WT	57.08	57.36	51.84	1.23
Eviscerated weight	515.93^a^	533.98^a^	427.67^b^	13.47
Breast weight	113.45^a^	119.24^a^	98.28^b^	3.04
Wing weight	73.61^a^	77.40^a^	63.01^b^	2.11
Drumstick weight	61.20^a^	62.08^a^	48.91^b^	1.71
Shank weight	20.80^a^	21.31^a^	11.82^b^	1.32
Thigh weight	76.18^a^	79.81^a^	63.90^b^	2.41
Head weight	30.84	31.32	30.65	0.50
Neck weight	32.77^a^	34.89^a^	28.11^b^	0.92
Dress weight	431.52^a^	446.46^a^	357.08^b^	11.45
Dress weight%	68.15	68.24	68.77	0.32
Organs yield Heart weight	3.00	3.24	2.78	0.10
Gizzard weight	22.46	21.74	20.25	0.49
Proventriculus weight	2.37	1.97	2.13	0.14
Crop weight	3.49	4.02	3.59	0.16
Lung weight	7.52	7.36	7.22	0.14
Small intestine weight	17.60	17.51	16.27	0.39

SEM: Standard error of the mean, GIT WT: Gastrointestinal tract weight.

Means with different superscripts on the same row differ significantly (*p *< 0.05).

#### Hot carcass weight

The CP levels in the feed had a significant (*p *> 0.05) impact on the mean hot carcass weight value, with T2 having the greater value of 511.83 gm and T3 (26%) having the lowest value of 647.83 gm, respectively. This is higher than the findings of Musundire [18].

#### Featherweight

The different CP treatments influenced the mean featherweight value in this study significantly (*p *> 0.05), with T2 recording the highest value of 56.50 gm and T3 recording the lowest value of 32.33 gm. These figures were lower than those reported by Kgakole et al. [19], who reported 93.14 gm for blue guinea fowl at 20 weeks, possibly due to age differences and the birds’ genetic makeup.

#### De-feathered weight

The de-feathered weight value was significantly *(p *> 0.05) influenced by dietary protein. T2 had the highest mean value of 591.33 gm, followed by T1, with a value of 573.00 gm, and T3, with the lowest value of 479.50 gm. These disparities could be attributable to dietary variances.

#### Gastrointestinal tract

The gastrointestinal tract weight was not significant (*p *> 0.05) in this study; hence, the different dietary protein treatments had no effect. However, T2 had the highest value of 57.36 gm, whereas T1 and T3 had 57.08 and 51.84 gm, respectively. The variances could be due to differences in nutrition and feed composition.

#### Eviscerated weight

The difference in protein levels in each treatment influences the mean value for eviscerated weight, which is significant (*p *> 0.05). T2 had the highest mean value of 533.98 gm, while T3 had the lowest value of 427.67 gm. This could be due to the bird’s body conformation.

#### Breast weight

The mean value was significant (*p *> 0.05). T2 had the highest value of 119.24 gm and T3 had the lowest value of 98.28 gm. These results are, however, lower than those observed by Chiroque et al. [17] and Kerketta and Mishra [20], who recorded 272 ± 21.07 and 259.33 ± 17.49 gm for pearl and lavender at 14 weeks. This could be attributed to the birds’ age and genetic makeup.

#### Wing weight

The difference in protein levels influences the mean value for wing weight. T2 had a better value of 77.40 gm, and T3 had the lowest value of 63.01 gm; these values were greater than those reported by Musundire [18], which were 53.5 and 55.4 gm, respectively. This variance could be due to the nutritional effect.

#### Drumstick weight

The dietary protein influenced the mean value of drumsticks, which was significant (*p *> 0.05). T2 had the greatest value of 62.08 gm, greater than Musundire’s [18] findings of 53.6 gm. This could be due to the impact of nutritional management.

#### Shank weight

The mean shank weight for this study was significant (*p *< 0.05). T2 had a higher value of 21.31 gm, higher than the findings of Kgakole et al. [19].

#### Thigh weight

The dietary protein levels influenced the mean value of thigh weight, which was significant (*p *< 0.05). T2 has the greatest mean value of 79.8 gm, followed by T1 with 76.10 gm and T3 with 63.90 gm, which were higher than 17.19 and 18.60 gm, recorded by Batkowska et al*.* [16] and Ahaotu et al. [21]. This could be due to the certainty that adequate dietary protein levels cause the birds to produce optimal yields.

#### Head weight

Dietary protein levels had no significant (*p *> 0.05) effect on the head weight mean value in this study. T2 had a better value of 31.32 gm, followed by T1 and T3, with relative values of 30.84 and 30.65 gm, respectively. These values were higher than those reported by Musundire [18] for mature male and female guinea fowls (26.3 and 27.3 gm). This can be the result of applying effective management strategies.

#### Dress weight

The dress weight is an essential indicator for assessing the quality of animal products. This study studied how dietary protein affected dress weight, and treatment T2 had the highest weight of 446.46 gm, followed by T1 with 431.52 gm and T3 with 436.46 gm. These values, however, were less than the 736.67, 700, and 708.33 gm, respectively, that Ebegbulem and Asuquo [3] recorded at week 14. The age of the sacrificed animals may be associated with the variations in dress weight values between the current study and the studies of Ebegbulem and Asuquo [3]. Animals tend to accumulate body fat as they become more mature, which could lead to higher dress weight values. As a result, the animals used in Ebegbulem and Asuquo’s [3] study were probably slaughtered at an older age than the birds used in this experiment.

#### Dress percentage

The dietary protein did not impact the dressing percentage in this study. T1 had a better value of 68.77%, while T3 had the lowest value of 68.15%. The results of this study were identical to those of Chiroque et al. [17]: 69.68% and 68.98%. This similar trend could be due to nutrition.

#### Organ yield

The internal organ indices in this study were not significantly (*p *> 0.05) impacted by dietary protein. The heart weight mean values were 3.24 gm in T2, 3.00 gm in T1, and 2.78 gm in T3. The results of Ahaotu et al. [21] for different dietary protein levels in guineas (3.89, 3.86, and 3.85 gm) were similar to those of this study. However, these are greater than the values of Batkowska et al. [16] at 14 weeks (0.64 and 0.64 gm). This could be due to genetic makeup. The greatest value for gizzard weight was 22.46 gm in T1, which was greater than the result of Houndonougbo et al*. *[22] for guinea fowl at week 16 but lower than the results of Yeboah et al. [23] for grower males and female guinea fowl at week 16. It might be possible that this was due to age differences. Furthermore, T1 had the highest mean value of 2.37 gm for proventriculus weight, while T2 (1.97 gm) had the lowest, possibly because of the feed absorption rate. T2 had the greatest crop weight mean value of 4.02 gm, whereas T1 had the lowest at 3.49 gm in this study.

## Conclusion

The dietary protein inclusion levels improved growth performance and carcass attributes. Guinea fowl (*N. meleagris*) fed a 24% dietary protein inclusion had the best growth performance, with a final mean weight of 619.83 gm; however, birds fed a 22% dietary protein had a similar final mean weight of 586.80 gm, while those fed 26% CP had the least final mean weight of 566.43 gm. Furthermore, birds fed 24% dietary protein had a greater mean weight throughout the treatments regarding carcass features. Even though the energy was isocaloric, the keets appeared to respond to the feed’s protein content. This study showed indigenous guinea fowls could not utilize excessively high CP levels in their diet. Farmers can feed a diet with 24% CP to indigenous guinea fowl, which has been proven to be the best for optimum performance and production in this study. Guinea fowl (*N. meleagris*) are known to be flighty and wild; hence, chicks should be raised in cages to avoid mortality due to stampedes. When cages are not readily available, the stocking density on the floor should be considered.

## List of abbreviations

%, percentage; CP, Crude protein; gm, gram; kcal/kg, kilo calorie per kilogram.
